# Strontium-Zinc conversion coating on magnesium plates for resorbable tack screws in guided bone regeneration: Characterization and biocompatibility evaluation

**DOI:** 10.4317/jced.62819

**Published:** 2025-08-01

**Authors:** Pradeed Kumar Yadalam, Parkavi Arumugam, Sai Keerthana Melanathuru-Balanatha, Carlos M Ardila

**Affiliations:** 1 Department of Periodontics, Saveetha Dental College, Saveetha Institute of Medical and Technology Sciences, SIMATS, Saveetha. University, Chennai, Tamil Nadu, India; 2 Department of Basic Sciences, Biomedical Stomatology Research group, Faculty of Dentistry, Universidad de Antioquia, U de A, Medellín, Colombia

## Abstract

**Background:**

Guided bone regeneration (GBR) requires resorbable implants that balance corrosion resistance and biocompatibility. Magnesium (Mg) is a promising candidate, but its rapid degradation necessitates protective coatings. This study develops and characterizes a strontium-zinc (Sr-Zn) conversion coating on Mg plates for resorbable tack screws, evaluating its corrosion resistance, surface properties, and biocompatibility.

**Material and Methods:**

Mg plates (20×15×2 mm) were etched with HCl, coated with Sr-Zn via immersion (30 min, pH 3–5), and characterized using SEM, EDX, and FTIR. Corrosion resistance was assessed via potentiodynamic polarization in simulated body fluid (SBF). Biocompatibility was evaluated using MG63 osteoblast cultures, with statistical comparison (Student’s t-test, *p* < 0.05) between coated (Group A) and uncoated (Group B) plates.

**Results:**

SEM revealed a dense, fibrous coating with interconnected pores, enhancing cell adhesion. EDX confirmed Zn (46.6 wt%) and Sr (3.7 wt%) incorporation. FTIR identified hydroxyl/carbonyl groups and metal-oxygen bonds. The coating improved corrosion resistance compared to bare Mg plates. In vitro cell culture assays demonstrated that Sr-Zn conversion-coated Mg plates (Group A) showed comparable cell viability to bare Mg plates (Group B) at all tested time points. Peak cell viability was recorded at 24 hours, with Group A achieving 92.66% and Group B reaching 91% (*p* = 0.238). This statistically non-significant difference suggests successful biocompatibility of the Sr-Zn coating. The enhanced biocompatibility observed is likely attributed to the coating’s improved corrosion resistance.

**Conclusions:**

The Sr-Zn coating improved Mg’s corrosion resistance while maintaining osteoblast viability, supporting its potential for resorbable GBR tack screws.

** Key words:**Guided bone regeneration, Magnesium implants, Strontium-zinc coating, Corrosion resistance, Biocompatibility, Resorbable screws.

## Introduction

Dental implants have become a cornerstone of modern dentistry, offering an optimal solution for tooth loss caused by trauma, decay, periodontal disease, or other etiologies. Their popularity stems from restored masticatory function, improved phonetics, enhanced aesthetics, and long-term patient satisfaction, significantly elevating quality of life. Recent innovations in implantology—such as digital treatment planning, guided surgery, surface modifications, zygomatic implants, and personalized approaches—have further optimized predictability and clinical outcomes [1]. However, successful implantation fundamentally depends on adequate bone volume and quality, which often necessitates regenerative interventions.

Guided bone regeneration (GBR) has emerged as a gold-standard technique to address bone deficiency, enabling implant placement in atrophic sites. This method utilizes barrier membranes, bone grafts, growth factors, and fixation devices like tack screws to achieve horizontal or vertical bone augmentation [2]. Resorbable and non-resorbable membranes, combined with autografts, allografts, xenografts, or synthetic grafts, are commonly employed alongside osteoinductive agents such as bone morphogenetic proteins (BMPs) and platelet-derived growth factors (PDGFs) to promote regeneration [3]. Crucially, tack screws stabilize these materials, particularly in anatomically complex regions [4]. Conventional titanium or stainless-steel screws, though effective, are prone to complications including palpability, mucosal exposure, infection, and MRI interference. Resorbable alternatives, notably magnesium (Mg)-based screws, have thus gained traction for mitigating these issues while reducing costs and patient morbidity [5].

Mg tack screws are particularly promising due to their biodegradability, bone-like mechanical properties, and biocompatible degradation products (e.g., Mg²+ ions) [6,7]. Their osteoinductive, antimicrobial, and anti-inflammatory effects further enhance regenerative potential. However, rapid corrosion in physiological environments remains a critical limitation. Degradation generates hydrogen gas (H2) and localized alkalization, which may lead to gas cavity formation, delayed osseointegration, and even tissue necrosis. For instance, rodent studies documented subcutaneous gas dispersion and reduced survival rates linked to uncontrolled H2 evolution [8].

To modulate corrosion rates, conversion coatings—such as magnesium fluoride (MgF2)—have been explored. These coatings reduce H2 emission and improve mechanical stability during degradation [5,9]. Building on this, we developed a strontium-zinc (Sr-Zn) conversion coating for Mg plates, aiming to synergize corrosion resistance with bioactivity. Sr and Zn were selected for their dual roles: Sr enhances osteogenesis, while Zn offers antimicrobial properties. This study characterizes the coating’s surface morphology, chemical composition, corrosion resistance, and biocompatibility, with the ultimate goal of optimizing resorbable tack screws for GBR applications.

## Material and Methods

The study was conducted at the Department of Biomaterials, Saveetha Dental College, Chennai, India. Mg plates used for conversion coating, with dimensions of 20×15×2 mm, were procured from a local supplier in Chennai, India. The chemicals required for the conversion coating process, including Sr and Zn salts, were analytical grade and sourced from Sigma-Aldrich, Merck Group, Darmstadt, Germany. For biocompatibility testing, osteoblast-like MG63 cells were obtained from the National Centre for Cell Science, Pune, India. The culture media, necessary supplements for cell culture, and reagents for cell staining were purchased from HiMedia Laboratories Private Limited, Thane, India.

Development of strontium-zinc conversion coating on magnesium

The Mg plates were cleaned by immersing them in ethanol and gently scrubbing with a soft brush to eliminate organic impurities. This was followed by a thorough rinse with deionized water to remove residual ethanol. For surface etching, the substrates were immersed in a 1 M hydrochloric acid (HCl) solution for 1 minute before rinsing with deionized water to neutralize the acid and remove any lingering salts. An additional ethanol rinse was performed to eliminate excess water, and the substrates were then dried using compressed air.

Solutions for the Sr-Zn conversion coatings were prepared. Zinc sulfate (ZnSO4) was dissolved in deionized water at a typical concentration of 0.1 M. Similarly, strontium chloride (SrCl2) was also dissolved in deionized water at a concentration of 0.1 M. The solutions were mixed in a 1:1 ratio of Sr to Zn for the Sr-Zn conversion coating bath. The pH of the conversion bath was adjusted between 3 to 5 using diluted phosphoric acid (H3PO4), and a sTable pH was maintained throughout the coating process to ensure consistent results.

The conversion coating process utilized an immersion technique wherein pretreated Mg plates were soaked in a conversion solution for 20-30 minutes to achieve a uniform coating thickness. The solution temperature was kept between 20-30°C to optimize the coating formation. Gentle agitation of the solution was applied to ensure consistent coating across the surface. After immersion, the Mg plates underwent thorough rinsing with deionized water, followed by ethanol rinsing, and were dried with compressed air. The samples were placed in a drying oven at 60-80°C for about one hour to ensure complete drying and stabilization of the coating.

The coatings were characterized for quality evaluation through Scanning Electron Microscopy (SEM), which examined surface morphology and uniformity. Energy Dispersive X-ray Spectroscopy (EDS) was utilized to verify the elemental composition, confirming the presence of elements such as Sr, Zn, and Mg. Fourier Transform Infrared (FTIR) spectroscopy analyzed the functional groups and chemical bonds within the conversion coating.

## Results

- Surface and Compositional Analysis

**Figure 1:**
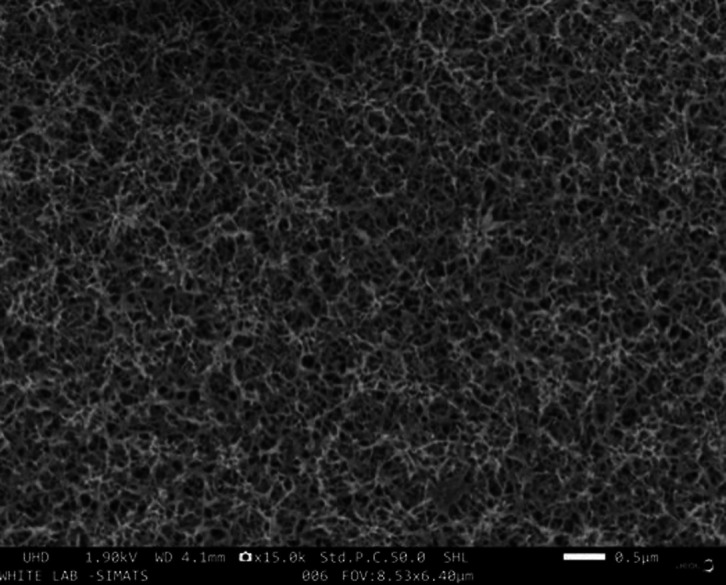
Surface characterization of Sr-Zn conversion coating on Mg plates.

**Figure 2:**
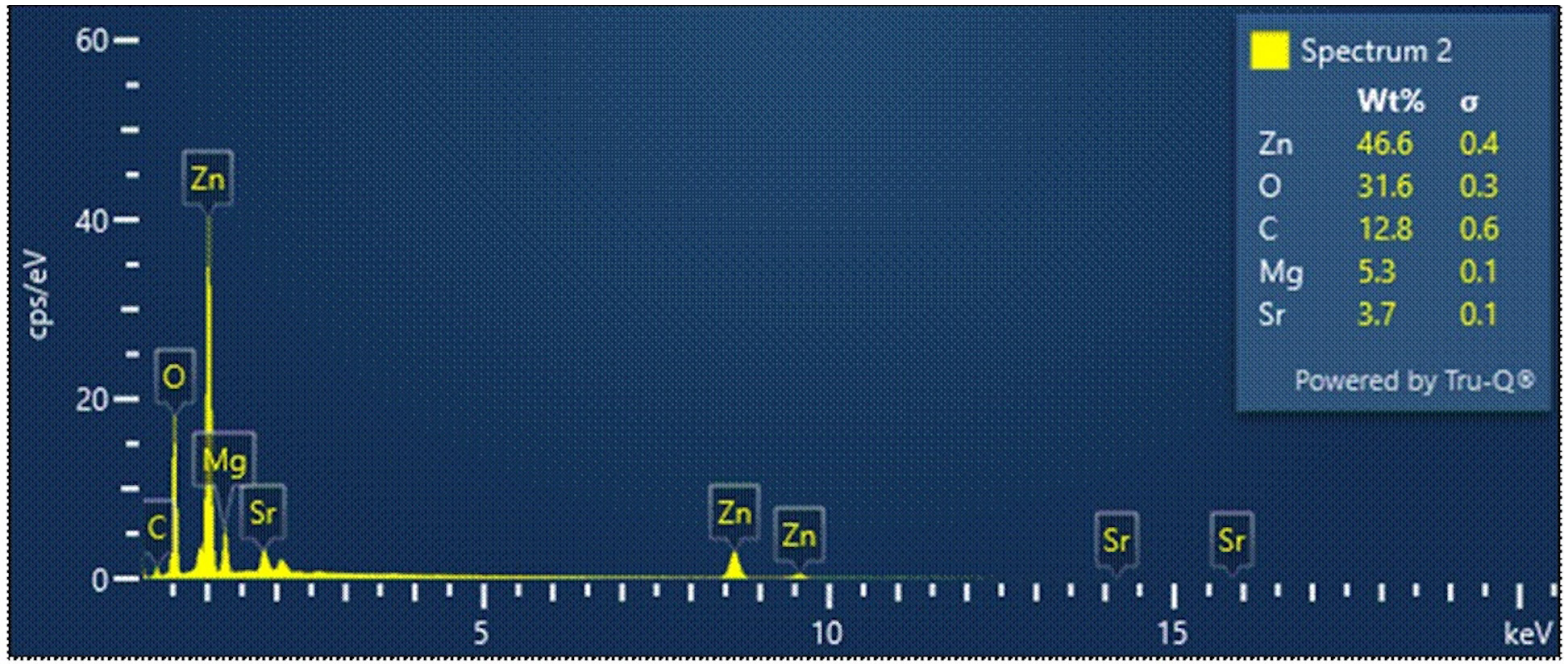
Elemental analysis of Sr-Zn conversion coating on Mg plates.

SEM revealed a dense, fibrous Sr-Zn coating uniformly distributed across the Mg surface (Fig. 1). The microstructure featured interconnected pores, which are advantageous for cell attachment and nutrient diffusion. EDS analysis confirmed the coating’s composition, with zinc as the predominant element (46.6 wt%), followed by oxygen (31.6 wt%), suggesting partial oxidation. Strontium was detected at 3.7 wt%, verifying its successful incorporation, while residual magnesium (5.3 wt%) and carbon (12.8 wt%) indicated minor substrate exposure and organic residues, respectively (Fig. 2). FTIR spectra ( Table 1) identified hydroxyl groups (O–H stretching, 3500–3600 cm-¹), likely from surface hydration, and carbonyl peaks (C=O, 1600 cm-¹), potentially attributable to residual ethanol. Metal-oxygen bonds (Zn–O and Sr–O) were observed between 400–700 cm-¹, confirming the coating’s inorganic framework.

- Corrosion Performance

**Figure 3:**
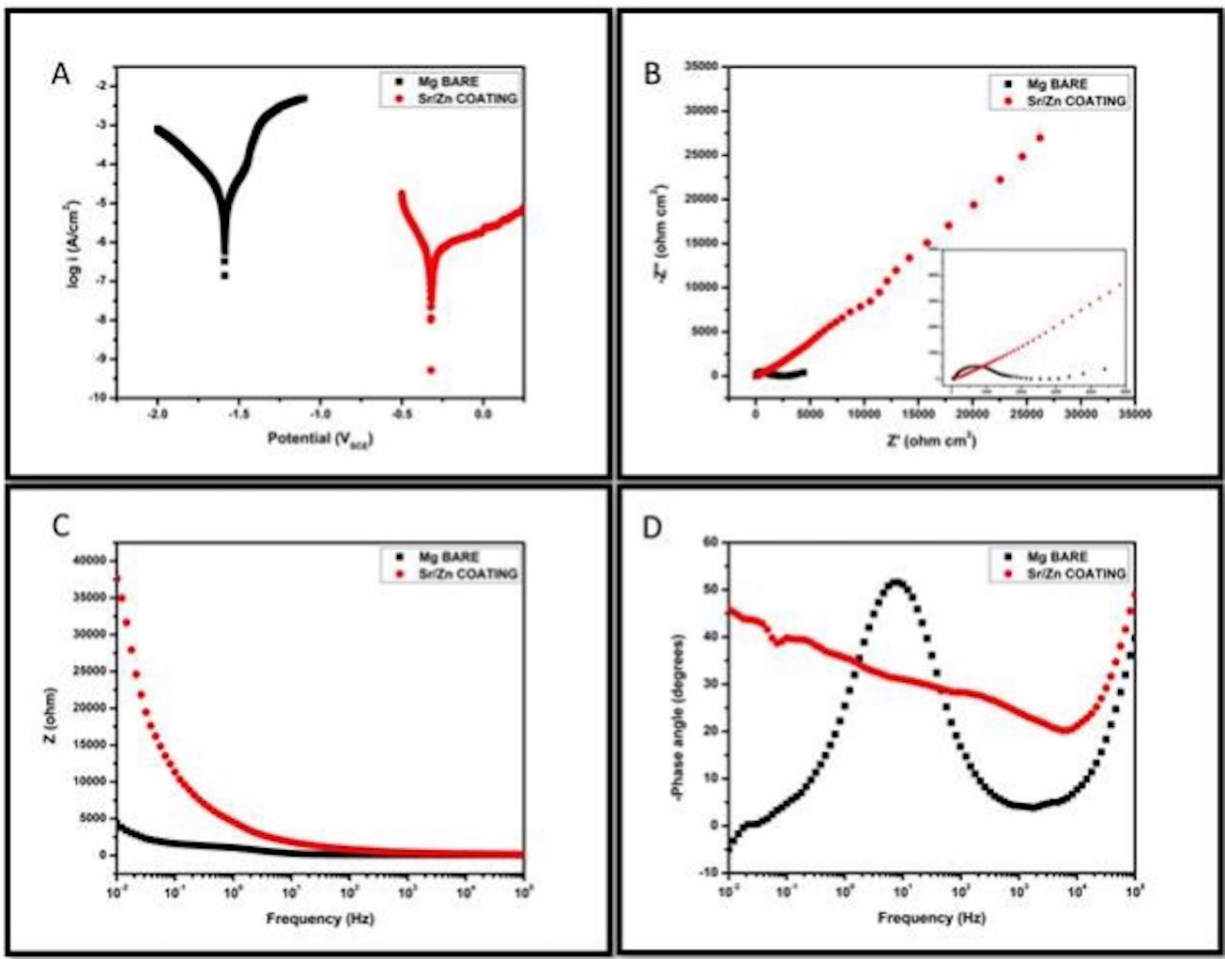
Corrosion analysis of Sr-Zn conversion coating on Mg plates.

Electrochemical testing demonstrated the coating’s protective efficacy. Tafel plots showed a significant reduction in corrosion current density (Icorr) for Sr-Zn-coated Mg compared to bare plates, indicating enhanced corrosion resistance. Nyquist and Bode plots further supported this, with larger semicircle diameters and higher impedance values for coated samples, reflecting improved charge-transfer resistance (Fig. 3).

- Biocompatibility Assessment

**Figure 4:**
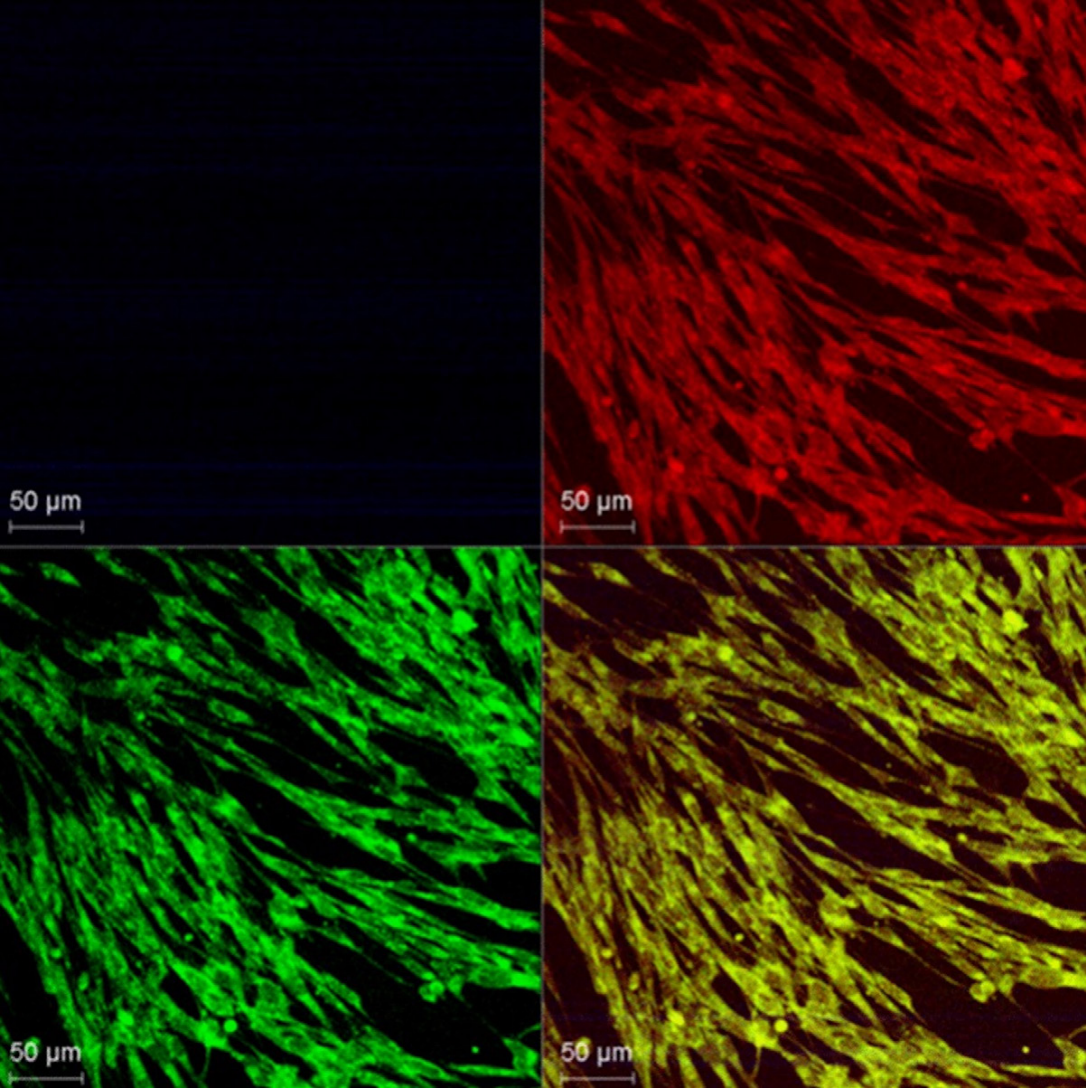
Confocal analysis of MG-63 cells cultured on Sr-Zn coated magnesium plates.

MG63 osteoblasts cultured on Sr-Zn-coated plates (Group A) exhibited spindle-shaped morphology with elongated filopodia, confirming active adhesion (Fig. 4). Cell viability at 24 hours was comparable between coated (92.66%) and uncoated (91%) groups (*p* = 0.238), with no statistical difference observed at later time points ( Table 2). Confocal imaging revealed multilayered cell growth and robust cytoskeletal organization, affirming the coating’s biocompatibility.

## Discussion

Recent advancements in regenerative dentistry and implantology have established guided bone regeneration (GBR) as a standard procedure for bone augmentation [10]. The widespread use of GBR membranes, bone grafts, and growth factors necessitates effective space maintenance and stabilization of these materials for successful outcomes. Tack screws have become indispensable in GBR techniques, providing enhanced stability and predictability for bone augmentation sites. However, the requirement for secondary surgery to remove titanium or stainless-steel screws increases patient morbidity. Additionally, infection prevention at the regenerative site remains crucial for procedural success. Magnesium (Mg) tack screws offer a biodegradable alternative, though their rapid initial degradation and associated hydrogen gas (H2) production can impair osseointegration. While few studies have explored Mg tack screws for regenerative applications, our research focuses on developing a strontium-zinc (Sr-Zn) conversion coating for Mg plates to enhance osseointegration, biocompatibility, and corrosion resistance while preserving biodegradability.

Material characterization confirmed successful application of the Sr-Zn conversion coating, which formed a uniform fibrous layer characteristic of such coatings. The fibrous structure with inherent porosity supports cell attachment and proliferation, promoting osseointegration. These findings align with studies of strontium phosphate Sr3(PO4)2 coatings on magnesium for mini-implant applications, which demonstrated consistent surface roughness, thickness, and adhesion strength [11]. Similar results were observed in hydrothermal-treated Sr3(PO4)2 coatings, where increased treatment temperatures yielded more compact coatings with improved corrosion resistance and bioactivity in Hank’s Balanced Salt Solution (HBSS). Such coatings show promise for various Mg-based medical devices, including cardiovascular stents and orthopedic screws.

Energy-dispersive X-ray spectroscopy (EDX) and Fourier-transform infrared spectroscopy (FTIR) analyses verified the Sr-Zn coating formation. Our FTIR results corroborate recent research on Sr3(PO4)2 coatings, which demonstrated reduced moisture permeability, homogeneous topography, and enhanced biocompatibility compared to uncoated Mg [12]. Comparable outcomes were reported for Sr-Zn coatings showing improved thermodynamic stability and corrosion resistance. Studies of strontium-calcium phosphate-coated magnesium (Sr-CaP-Mg) revealed superior corrosion resistance, cell proliferation, and bone formation, making it suitable for dental and orthopedic applications [13]. Zinc oxide (ZnO) coatings on Mg alloys via physical vapor deposition similarly enhanced osseointegration and corrosion resistance while reducing current density [14]. Multifunctional polypyrrole/ZnO composite coatings on Mg alloys demonstrated remarkable antibacterial activity against *Escherichia coli* while promoting cell adhesion [15]. Additional studies confirmed that silver-doped ZnO coatings on Mg alloys provided superior corrosion protection in simulated body fluid (SBF) [16], while ZnO films on AZ31B magnesium alloy showed enhanced antibacterial properties and cell proliferation [17].

Biocompatibility analysis revealed comparable cell viability between coated and uncoated Mg plates, confirming the coating’s safety. These findings align with research on zinc-incorporated titanium dioxide (TiO2) coatings, which enhanced adhesion, proliferation, and differentiation of bone marrow stem cells while inhibiting *Staphylococcus aureus* and *E. coli* [18]. The observed biocompatibility may stem from Zn ion release, consistent with studies demonstrating Sr and Zn’s ability to enhance osseointegration through antimicrobial and antioxidant properties [19].

Strontium has gained attention for osteoporosis treatment due to its dual action: promoting osteoblast proliferation while inhibiting osteoclast activity. It disrupts ruffled border formation in pre-osteoclasts, limiting resorption capacity [20], while enhancing calcium-sensing receptor activity to facilitate osteogenesis. Titanium micro/nano structures incorporating strontium (SLA-Sr) increased angiogenic capacity in human umbilical vein endothelial cells by upregulating hypoxia-inducible factor 1-alpha (HIF-1α) and extracellular signal-regulated kinase 1/2 (Erk1/2) phosphorylation [21]. SLA-Sr also promoted M2 macrophage polarization and pro-angiogenic platelet-derived growth factor secretion. Other studies highlight the osteogenic potential of Sr-doped calcium phosphate scaffolds and Sr-infused hydroxyapatite (HA) at bone-implant interfaces [21,22]. Local Sr administration at fracture sites has shown promise for bone healing in osteoporotic conditions [22], with systematic reviews supporting Sr’s role in improving implant osseointegration [23]. Additionally, Sr exhibits antimicrobial properties through multiple mechanisms, including cell membrane disruption and biofilm inhibition, particularly against S. aureus and *E. coli* [24].

Zinc-rich coatings demonstrate improved biocompatibility, corrosion resistance, and antibacterial properties while enhancing osseointegration. Zinc regulates osteoblast differentiation through Runt-related transcription factor 2/core-binding factor subunit alpha-1 (Runx2/Cbfa1) and Osterix gene expression. It activates bone morphogenetic protein 2 (BMP-2) signaling and influences bone mineralization by stabilizing calcium precursors within collagen fibrils [25]. Zinc supplementation increases alkaline phosphatase (ALP) activity and osteocalcin levels in ovariectomized rats [26], while zinc-doped carbon dots enhance osteogenic activity and bone formation *in vivo* [27]. Zinc also protects osteoblasts from oxidative stress-induced apoptosis by inhibiting cytochrome-C release and reducing p38 mitogen-activated protein kinase (P38) and c-Jun N-terminal kinase (JNK) phosphorylation [25]. On titanium implants, zinc coatings modify cellular microenvironments to enhance adhesion and promote pro-healing macrophage polarization.

Zinc’s primary antimicrobial mechanism involves ion release causing bacteriolysis and oxidative damage to bacterial components [28]. Zinc ions interact with bacterial membranes, altering charge balance and causing cellular deformation [29]. They induce conformational changes in bacterial enzymes, reversibly inhibiting activity while maintaining long-term antibacterial effects through sustained ion release. Systematic reviews confirm the bactericidal efficacy of zinc-based coatings on titanium implants [30].

Sr-Zn conversion coatings on Mg plates synergistically enhance osseoinduction, corrosion resistance, and antibacterial properties. While Mg degradation occurs through corrosion, the Sr-Zn coating optimizes degradation rates by slowing corrosion and reducing initial H2 production, allowing sufficient time for bone graft integration. The coating’s osseoinductive properties stabilize GBR tack screws during bone remodeling, while its antibacterial effects prevent graft site infections, collectively improving clinical outcomes.

This study has limitations, including unassessed mechanical properties, antimicrobial efficacy, mineralization potential, and degradation rates of the Sr-Zn coating. While results demonstrate biocompatibility and corrosion resistance, further cell line and *in vivo* studies are needed to evaluate clinical efficacy for tack screw applications.

## Conclusions

The Sr-Zn conversion coating on Mg plates presents a promising approach for fabricating GBR tack screws with enhanced osteoinductive properties, corrosion resistance, and antibacterial effects. By optimizing the degradation rate of Mg screws, this coating ensures adequate time for bone graft integration while preventing infection and improving stabilization during augmentation procedures.

## Figures and Tables

**Table 1:** FTIR analysis of Sr-Zn conversion coating on Mg plates.

Wave number (cm-1)	Bond	Functional group
3600 - 3500	-OH	Hydroxyl group
1600	C=O stretching vibrations Interlayer water molecules	Carbonyl group Hydroxyl group
500 - 700	Metal interaction oxygen Zn-O stretching vibrations Sr-O stretching vibrations	Oxide
400 - 500	Mg-O stretching vibrations	oxide

**Table 2:** Biocompatibility analysis of the Sr-Zn coated magnesium implants in comparison with bare magnesium implants – Student’s t test.

Time	Sample	n	Mean (% of cell viability)	Standard deviation	mean difference	p value	95% confidence interval	
							lower limit	upper limit
24 hours	group a	3	92.66	2.516	1.666	0.238	89.813	95.507
	group b	3	91	1.000			89.868	92.132
48 hours	group a	3	89.33	2.516	2.666	0.812	86.483	92.177
	group b	3	86.66	2.081			84.305	89.015
72 hours	group a	3	87.000	1.000	2.000	0.422	85.868	88.132
	group b	3	85.000	2.000			82.737	87.263
96 hours	group a	3	84.666	2.081	2.666	0.587	82.305	87.015
	group b	3	82.000	2.645			79.007	84.993
120 hours	group a	3	80.333	1.527	3.000	0.519	78.602	82.058
	group b	3	77.333	2.081			74.975	79.685

## Data Availability

The datasets used and/or analyzed during the current study are available from the corresponding author.
